# Impact of Preoperative Patient Education on Postoperative Recovery in Abdominal Surgery: A Systematic Review

**DOI:** 10.1007/s00268-022-06884-4

**Published:** 2023-01-15

**Authors:** Freya Brodersen, Jonas Wagner, Faik Güntac Uzunoglu, Corinna Petersen-Ewert

**Affiliations:** 1 Department of General-, Visceral-and Thoracic Surgery, University Medical Center Hamburg-Eppendorf, Martinistrasse 52, 20246 Hamburg, Germany; 2 Department Nursing and Management, University of Applied Sciences, Alexanderstrasse 1, 20099 Hamburg, Germany

## Abstract

**Background:**

Patient education is recommended as an essential component of Enhanced Recovery after Surgery (ERAS) protocols. However, there are many uncertainties regarding content and methodological criteria, which may have a significant impact on the effectiveness of the intervention. The aim of this review is to assess the effect of preoperative patient education on postoperative recovery in abdominal surgery and to examine different patient education strategies for their effectiveness.

**Methods:**

We performed a systematic review according to the PRISMA guidelines. PubMed, CINAHL, and Cochrane were searched from 2011 to 2022. All studies investigating the effect of preoperative patient education on postoperative recovery in abdominal surgery were included. A critical quality assessment of all included studies was performed.

**Results:**

We identified 826 potentially suitable articles via a database search and included 12 studies in this review. The majority of the included studies reported a reduction in the length of hospital stay (LOS) and even a reduction in postoperative complications and adverse events. Patients with preoperative education seemed to have lower psychological stress and experience less anxiety. However, the contents, delivery, and general conditions were implemented differently, making comparison difficult. Moreover, the majority of the included studies were weak in quality.

**Conclusion:**

With this review, we report potential effects, current implementations, and frameworks of patient education. However, the results must be interpreted with caution and are not directly transferable to clinical practice. Further studies in this field are necessary to make concrete recommendations for clinical practice.

## Introduction

Disease management programs (DMPs) for patients with chronic conditions have been established in clinical practice. An integral part of these programs is the education of patients regarding health-promoting behaviors and adherence to medical interventions and therapies [1–5]. DMPs positively affect the health-related quality of life, coping status, and self-management skills of patients [2, 4]. In line with DMPs, preoperative patient education is recommended as an essential part of Enhanced Recovery after Surgery (ERAS®) protocols. The objective of ERAS® pathways is to improve and accelerate recovery from surgery through evidence-based treatment [6, 7]. Empowering patients to take an active role in their treatment is highly relevant according to ERAS® concepts. Therefore, patient education is needed to ensure participation from the beginning of treatment.

While the benefits of preoperative patient education have been extensively studied for cardiac and orthopedic surgery, research is needed for abdominal surgery [7–10]. It seems evident that the content and didactic methods must differ in various surgical fields. Depending on the surgical procedure, multiple consequences ensue in the daily life of the patients and their relatives. Therefore, educational intervention must go beyond simply providing information to actually impacting patients' behavioral levels [11–14]. It is precisely this criterion that is not fulfilled in many surveys and thus, causes recommendation bias. Accordingly, the level of evidence in the ERAS® guidelines for preoperative patient education is estimated to be "low" with nevertheless a high recommendation rate for clinical practice [10–12]. It is unclear which strategies, outcome parameters, contents, and framework are appropriate for preoperative patient education. Ronco and colleagues published a systematic review exploring the strategies and benefits of patient education, across various surgical fields [13]. During the digitalization of the health care system and progressive development of new technologies, it can be assumed that new strategies are being used today to train patients before surgery. Hence, a systematic review is needed to analyze the current state of research regarding abdominal surgery. This systematic review aims to evaluate the impact of preoperative patient education on recovery after abdominal surgery and to examine strategies of patient education for their effectiveness.

## Methods

We conducted a systematic review using the preferred reporting elements for systematic reviews and meta-analyses (PRISMA) [14]. First, we applied the PICO (population, intervention, control and outcome) scheme to create an appropriate research question (Table 1). As a result, we primarily assessed two questions:

**Table 1:** PICO elements for creating a research question

Population	Intervention	Control	Outcome
Patients undergoing surgery with abdominal approach Including: Minimally invasive or open surgery	Patient education prior surgery Including: Any educational strategy used prior to surgery	No education, Routine practice, written pamphlets, comparable interventions	Recovery Including: Length of stay, complications, behavioral skills, coping ability, emotional or wellbeing status

First, what is the impact of preoperative patient education on recovery in abdominal surgery? Second, which strategies are used to train patients prior to abdominal surgery?

Since there is a vast variety of definitions for patient education, we formulated an appropriate definition for our review. This was necessary to ensure that education is meant as a planned and goal-directed intervention that does not only target increasing patients’ knowledge of specific topics. In the context of this study, we defined patient education as follows:

Patient education is a systematically planned and organized learning experience to achieve voluntary behavioral improvement based on increased knowledge and empowerment of the patients [15–18].

At least one of the following criteria to fulfill this definition must be met:

1. Educational intervention focuses on health literacy and behavioral or emotional skills
2. Educational intervention is based on a didactic concept or strategy
3. Educational intervention is patient-centered

### Research strategy and selection criteria

We searched the PubMed, CINAHL, and Cochrane databases for the period from 2011 to 2022. The entire database search took place in November 2021 and was conducted again in April 2022 to include new publications. The systematic literature research, data collection, and critical quality appraisal were conducted independently by two authors (FB, JW) and supervised by a third author (CPE). In case of disagreements, a re-evaluation was performed, and consensus was reached by consulting further independent authors. No automation tools were used within this review. The research was performed using the following terms: prior surgery OR preoperative; patient education OR patient education as topic [MeSH Terms] OR patient counseling; visceral surgery OR abdominal surgery OR general surgery. Synonyms or relative terms were related to the Boolean operator “OR,” and each set of topics was linked with the Boolean operator "AND." The results were limited to studies in German and English language and research with adult humans. Reference lists from all included trials were searched for further eligible studies (FB, JW). All studies focusing on preoperative counseling and educational concepts for patients were included. Multimodal prehabilitation concepts with an educational focus were also included. The study design was not limited, but comments or expert opinions, as well as unsystematic reviews, were excluded from this research. The setting was elective surgery in the field of abdominal surgery. Studies addressing ERAS implementation strategies or feasibility, educational programs for parents with children undergoing surgery, educational concepts for ambulatory surgery, and risk prediction tools were excluded. Systematic reviews and meta-analyses were included if at least one of their examined studies dealt with abdominal surgery.

### Data collection and critical appraisal

At the beginning of the study selection, we screened all titles of the studies for eligibility. If inclusion or exclusion criteria were disputed, the study was initially included. After exclusion of duplicates, the same procedure was performed with the remaining abstracts, followed by full-text screening. For data collection, we used standardized data extraction forms created with SRDR + (FB). A pilot test was performed (JW). Data extraction focused on the setting, delivery, timing, content, method, material, and outcome of the patient education. We were open to various outcome parameters, but the established criteria were measurement within the postoperative period and patient-centered outcomes for recovery. This means that studies exploring cost-effectiveness or only satisfaction were excluded. A critical quality appraisal of the studies was performed using RoB 2 [19], AMSTAR 2 [20], or ROBINS-I [21], depending on the study design (FB, JW).

## Results

### Study characteristics

**Fig. 1:**
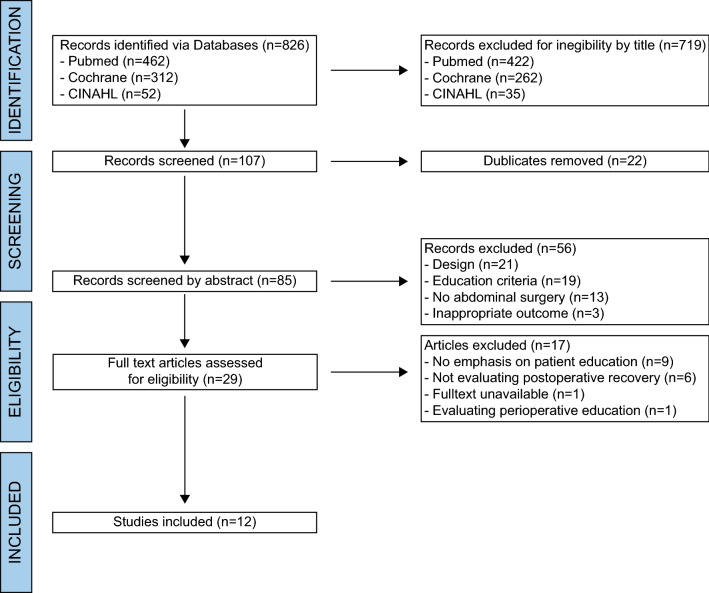
PRISMA Flowchart

The systematic search yielded 826 literature results (Fig. 1). Seven hundred and nineteen articles were excluded because the titles of the articles were not suitable for this research. Twenty-two duplicates were removed. Accordingly, we reviewed 85 abstracts for their suitability and excluded 56 publications. We screened 29 full-text and performed a critical appraisal. Twelve Studies were included in this review (Table 2). The quality of six included studies was estimated as low (FB, JW) [13, 24, 25, 29, 31, 32]. Three studies were rated as medium quality [23, 26, 30] and three as high [22, 27, 28]. The details on risk of bias assessment and quality appraisal of the included studies are found in Table 3. Table 4 shows detailed reasons for excluding studies after full-text screening [33–49]. The reason for exclusion was a lack of focus on education in most studies. Within the included studies were four randomized controlled trials [22–25], and four systematic reviews, of which two included a meta-analysis [13, 26–28], and four were Non-Randomized Studies of Intervention (NRSIs) [29–32]. Freeman et al. (2018) provided a reanalysis of a recent meta-analysis [27, 28]. Both reviews were included. The included studies came from a wide variety of countries: three from the United Kingdom, two from Turkey, and one each from Australia, China, Germany, Italy, Korea, Spain, and USA. The studies in different surgical fields ranged from minimally invasive cholecystectomies to bariatric and colorectal surgery and extended major abdominal surgery [24–26, 29, 30].

**Table 2:** Characteristics of included studies

References	Study design	Area of surgery	n	Educational intervention	Control	Outcome	Critical quality appraisal
Barberan-Garcia et al. [22]	RCT	Major abdominal surgery	125	Personalized prehabilitation, including Motivational interviews	Standard care	LOS, complication, severity of complication, endurance time, mobility, physical activity, quality of life, anxiety	High quality
Brown et al. [29]	NRSI Observational study	Bariatric surgery	143	Educational package with interactive e-learning-modules	Standard care	Excess weight loss (%), participation rate, preoperative weight change, surgical complications, fail to attend rate	Low quality
Çakır and Özbayır [24]	RCT	Colorectal surgery	60	Education for stoma care	Standard care	anxiety	Low quality
Cavallaro et al. [30]	NRSI Cohort Study	Colorectal surgery	505	Educational phone call about ERAS	Standard care	LOS, readmission, complications,	Medium quality
Freeman et al. [27]	Meta analysis	Surgery under general anesthesia	n.a.	Psychological Preparation techniques	Standard care	LOS, pain, negative affect,	High quality
Gurusamy et al. [26]	Systematic review	Laparoscopic Cholecystectomy	431	Preoperative educational strategies	Standard care	Mortality, Morbidity, quality of life, LOS, patient knowledge, pain, patient satisfaction	Medium quality
Hong and Lee [31]	NRSI Cohort Study	Gynecological surgery	79	Education for use of patient-controlled analgesia (PCA)	Standard care	Pain, adverse reaction, knowledge and attitude towards PCA, patient satisfaction, cumulative infused dose of analgesics	Low quality
Klaiber et al. [25]	RCT	Major abdominal surgery	244	Group education	Standard care + written pamphlet	LOS, complications, serious adverse events (SAE), 30-day mortality, pain, anxiety, quality of life, patient satisfaction, feasibility of cluster randomization	Low quality
Peng et al. [23]	RCT	cholecystectomy	217	anesthesia education via ASP (Anesthesia service Platform)	Standard care	LOS, pain, anxiety, wellbeing, 5 most frequently asked questions	Medium quality
Powell et al. [28]	Meta analysis	Surgery under general anesthesia	10302	Psychological preparation techniques	Standard care	LOS, pain, behavioral recovery, negative affect,	High quality
Ronco et al. [13]	Systematic review	All fields of surgery	3944	Educational interventions prior surgery	Standard care	LOS, anxiety, satisfaction, depression, quality of life, health status, self-efficacy, emotional wellbeing	Low quality
Soydaş and Yildiz [32]	NRSI Cohort study	Abdominal surgery	35	Watching educational video	Standard care	Anxiety, satisfaction	Low quality

**Table 3:** Critical Quality Appraisal

References	Quality appraisal tool	Main domains for critical appraisal
Barberan-Garcia et al. [22]	ROB 2	**R:** low risk of bias
		**D:** some concerns
		**Mi:** low risk of bias
		**Me:** low risk of bias
		**S:** low risk of bias
		**O:** low risk of bias
Brown et al. [29]	ROBINS-I	**Confounding**: serious risk of bias
		**Selection of participants**: moderate risk of bias
		**Classification of interventions**: low risk of bias
		**Deviations from intervention**: critical risk of bias
		**Missing data**: moderate risk of bias
		**Outcome measurement:** moderate risk of bias
		**Reported results:** moderate risk of bias
Çakır SK, Özbayır T [24]	ROB 2	**R:** high risk of bias
		**D:** high risk of bias
		**Mi:** some concerns
		**Me:** some concerns
		**S:** high risk of bias
		**O:** high risk of bias
Cavallaro et al. [30]	ROBINS-I	**Confounding**: serious risk of bias
		**Selection of participants**: critical risk of bias
		**Classification of interventions**: serious risk of bias
		**Deviations from intervention**: low risk of bias
		**Missing data**: No information
		**Outcome measurement:** low risk of bias
		**Reported results:** low risk of bias
Freeman et al. [27]	AMSTAR II	**Registered protocol**: n.a
		**Adequate literature research:** yes
		**Justification for exclusion:** yes
		**Risk of bias assessed:** n.a
		**Appropriate meta-analysis:** yes
		**Consideration of bias for interpretation of results:** yes
		**Assessment of publication bias:** no
Gurusamy et al. [26]	AMSTAR II	**Registered protocol**: yes
		**Adequate literature research:** partial yes
		**Justification for exclusion:** partial yes
		**Risk of bias assessed:** yes
		**Appropriate meta-analysis:** no
		**Consideration of bias for interpretation of results:** yes
		**Assessment of publication bias:** no
Hong and Lee [31]	ROBINS-I	**Confounding**: critical risk of bias
		**Selection of participants**: critical risk of bias
		**Classification of interventions**: moderate risk of bias
		**Deviations from intervention**: low risk of bias
		**Missing data**: No information
		**Outcome measurement:** critical risk of bias
		**Reported results:** low risk of bias
Klaiber et al. [25]	ROB 2	**R:** low risk of bias
		**D:** high risk of bias
		**Mi:** high risk of bias
		**Me:** some concerns
		**S:** high risk of bias
		**O:** high risk of bias
Peng et al. [23]	ROB 2	**R:** some concerns
		**D:** some concerns
		**Mi:** high risk of bias
		**Me:** some concerns
		**S:** low risk of bias
		**O:** high risk
Powell et al. [28]	AMSTAR II	**Registered protocol**: yes
		**Adequate literature research:** yes
		**Justification for exclusion:** yes
		**Risk of bias:** yes
		**Appropriate meta-analysis:** yes
		**Interpretation of results:** yes
		**Assessment of publication bias:** no
Ronco et al. [13]	AMSTAR II	**Registered protocol**: partial yes
		**Adequate literature research:** partial yes
		**Justification for exclusion:** no
		**Risk of bias:** no
		**Appropriate meta-analysis:** n.a
		**Consideration of bias for interpretation of results:** no
		**Assessment of publication bias:** no
Soydaş and Yildiz [32]	ROBINS-I	**Confounding**: critical risk of bias
		**Selection of participants**: critical risk of bias
		**Classification of interventions**: moderate risk of bias
		**Deviations from intervention**: low risk of bias
		**Missing data**: No information
		**Outcome measurement:** critical risk of bias
		**Reported results:** moderate risk of bias

**Risk of bias legend (ROB 2)**

**R:** Bias arising from the randomization

process **D:** Bias due to deviations from the intended intervention

**Mi:** Bias due to missing outcome data

**Me:** Bias in measurement of the outcome

**S:** Bias in selection of the reported results

**O:** Overall risk of bias

**Table 4:** Excluded studies after full-text screening

References	Title	Reason for exclusion
Cavalheri and Granger [33]	Preoperative exercise training for patients with non-small cell lung cancer	Does not meet educational criteria, evaluating simply exercise training prior surgery
Elhage et al. [34]	Preoperative patient opioid education, standardization of prescriptions, and their impact on overall patient satisfaction	Evaluating satisfaction with pain management, postoperative recovery is not focused no participatory component
Fenton et al. [35]	Prehabilitation exercise therapy before elective abdominal aortic aneurysm repair	evaluating prehabilitation prior surgery, no participatory component, no education is in detail described
Forsmo et al. [36]	Compliance with enhanced recovery after surgery criteria and preoperative and postoperative counseling reduces length of hospital stay in colorectal surgery: results of a randomized controlled trial	Evaluating ERAS Implementation within colorectal surgery, no emphasis on education, no evaluation of the educational aspect
García-Delgado et al. [37]	Prehabilitation for Bariatric Surgery: A Randomized, Controlled Trial Protocol and Pilot Study	Evaluation of preoperative physical activity and respiratory muscle training, no evaluation of the educational aspect
Howard et al. [38]	Taking Control of Your Surgery: Impact of a Prehabilitation Program on Major Abdominal Surgery	No educational focus, evaluating prehabilitation, no education focused outcome parameters
Huber et al. [39]	Multimedia support for improving preoperative patient education: a randomized controlled trial using the example of radical prostatectomy	Evaluation the surgical information prior surgery and satisfaction with surgical education, educational focus is on knowledge gain and decision making, and recovery is not focused
Lin et al. [40]	The effect of an anaesthetic patient information video on perioperative anxiety: A randomised study	Evaluating Anxiety and satisfaction, watching video vs. standard information, educational criteria are not met
Loughney et al. [41]	Exercise interventions for people undergoing multimodal cancer treatment that includes surgery	Only evaluating exercise training no educational focus
Pandrangi et al. [42]	The Application of Virtual Reality in Patient Education	VR used additional to surgical information prior surgery; postoperative recovery not focused
Priya and Roach [43]	Effect of preoperative instruction on anxiety among women undergoing abdominal hysterectomy	No full-text available, request send to the authors
Sheaffer et al. [44]	Decreasing length of stay in bariatric surgery: the power of suggestion	Does not meet the education criteria. Evaluating patient’s expectations in relation to LOS
García-Botello et al. [45]	Implementation of a perioperative multimodal rehabilitation protocol in elective colorectal surgery. A prospective randomized controlled study	Only evaluation of a fast-track concept, no educational component
Teishima et al. [46]	Usefulness of personalized three-dimensional printed model on the satisfaction of preoperative education for patients undergoing robot-assisted partial nephrectomy and their families	Does not meet the education criteria no focus on postoperative recovery
Wall et al. [47]	Strength Training Enhances Recovery After Surgery (STERAS)	No educational focus, no education described
West et al. [48]	The effects of preoperative, video-assisted anesthesia education in Spanish on Spanish-speaking patients' anxiety, knowledge, and satisfaction: a pilot study	Focus is on language barriers not on recovery
Zhang et al. [49]	Perioperative comprehensive supportive care interventions for chinese patients with esophageal carcinoma: a prospective study	Focus on pre and postoperative Education and supportive Intervention, no single evaluation of preoperative education

## Outcomes

Within the clinical trials in this review, a total of *n* = 1.554 patients were included, of whom *n* = 732 were in the intervention groups and *n* = 822 were in the control groups. The four systematic reviews included a total of *n* = 14.677 patients. Length of hospital stay (LOS) and postoperative morbidity were the most reported outcome parameters in the studies [13, 22, 23, 25–28, 30]. Most studies reported a significant reduction in LOS within the intervention groups [22, 23, 27, 28, 30]. Only one study did not show a difference in length of stay [25]. Eight studies reported postoperative morbidity and adverse events [13, 22, 23, 25, 26, 29–31]. Barberan-Garcia et al. showed significantly lower complication rates in the intervention group but no differences in the severity of complications [22]. Klaiber et al. reported significantly lower in-hospital falls in their education group [25]. Cavallaro et al. had fewer surgical site infections in the education group, but the difference was not statistically significant [30]. Hong et al. reported fewer adverse events (dizziness) during the use of patient-controlled analgesia (PCA) within the educational group [31].

Psychological status was commonly reported [13, 22–28, 32]. Three studies underlined a reduction in anxiety with patient education. [23, 24, 32]. Two systematic reviews stressed a reduced negative affect with psychological preparation, with procedural information appearing to be most effective [27, 28]. However, two further studies did not show a difference between their groups in terms of anxiety [22, 25]. Postoperative pain was reported in six included studies with conflicting results [23, 25–28, 31]. Freeman et al. and Powell et al. demonstrated a significant improvement in postoperative pain, not only with the teaching of relaxation techniques but also with the combination of behavioral instruction and sensory information [27, 28]. Hong et al. reported significantly lower pain in their education group [31]. Two studies did not find statistically relevant differences in this area [25, 26]. Peng et al. detected even higher postoperative pain levels in their intervention group [23]. The Impact on quality of life (QoL) was examined in three studies [13, 22, 25]. None of the studies reported differences in QoL within intervention or control groups.

## Contents

The content of patient education varied widely from very general in some studies to very specific to the particular procedure in others. All studies addressed preparation for surgery, but with different approaches. Recommendations and guidance on preoperative physical activity or postoperative mobilization were the most frequently mentioned patient education content [22, 29, 30, 32]. Instructions and advice on respiratory therapy were also frequently mentioned as comprising the content of preoperative patient education [25, 32]. This frequency was followed by that of nutritional counseling [22, 29, 30] and psychological preparation (motivation, stress reduction) [25, 27–29]. In three of the included studies, pain management was also a criterion of content within the education [13, 25, 31]. Adherence to medical therapies or interventions, for example, the intake of medication, was also part of two studies [30, 31]. Patients were informed about structural processes in the hospital [13, 32] and postoperative complications [13, 25]. Regarding colonic surgery, stoma care was addressed in two of the included studies [13, 24]. Most of the education took place in individual sessions, and only Klaiber et al. conducted group settings [25]. In the majority of studies, the education was delivered face to face [22, 24, 25, 31, 32]. Some studies provided education via websites, e-training or videos [23, 29, 31, 32]. Written pamphlets were additionally provided in four studies [24, 25, 30, 31]. In one study, patients were trained via phone call [30]. In most cases, preoperative patient education was provided by nurses [24, 25, 30, 32]. In one study, patient education was provided by an anesthesiologist, and in another study, education was provided by a physiotherapist [22]. The timing of education varied widely across the included studies, that is from earlier than 4 weeks before surgery [22, 29] to one day before surgery [24, 25, 31].

## Discussion

Abdominal surgery is associated with high morbidity and mortality [22]. Measures such as patient education are needed to improve recovery after surgery.

In this systematic review, we assessed the effects of patient education on postoperative recovery in abdominal surgery. In many areas, preoperative patient education seems to positively impact the postoperative course, especially length of stay, postoperative adverse events, and psychological status [22–25, 27, 28, 30, 31]. The outcome criteria investigated differed significantly among the studies examined. Transparent cause–effect relationships cannot be established for patient education since a wide range of effects can be expected. Measuring these factors require more research at the emotional, behavioral, cognitive, and participatory levels.

High heterogeneity in the delivery and setting of preoperative patient education across the intervention groups was evident. Moreover, the timeframes varied from days to several weeks before surgery. The timing of education may be crucial for patients to develop coping strategies and take an active role in the treatment process for the patient, but this aspect was not critically questioned in any study. None of the included studies defined preoperative patient education or which criteria must be met. Furthermore, the setting and strategy of patient education were poorly described. We hardly found written educational concepts or strategies; at best, lists with educational contents were presented. Only two studies reported that the education followed a manual or written protocol [24, 30]. To enable quality criteria and comparability, it is advisable to define a didactic concept or strategy to facilitate sustainability and verifiability of the outcomes. In particular, conversation techniques or conversation styles, as well as didactic methods, were rarely described. Only one study described the interview style consisting of Motivational Interviewing (MI) [22]. No qualitative studies of preoperative patient education in abdominal surgery examined patient needs or experiences. Studies in this area are needed to ensure patient-centeredness and need-based education.

The treatment of the control groups was poorly described in most studies. Thus, patients in the control group were often reported as receiving a “standard treatment” without specifying what this included. Since "standard care" already varies from setting to setting, it is impossible to derive comparability without a detailed description of such treatment.

We estimated the overall quality in most included studies as ranging from low to medium. In various studies, we noticed a high risk of selection bias and a nontransparent study process [24, 25, 29, 30]. Due to the nature of the intervention, blinding of the patients was impossible, but the treatment providers were often not blinded. Performance bias may have occurred in the studies examined due to more intensive care in the educational populations. This means that positive effects may have been due to the more intensive care and not to the education. This consideration is not reflected in any of the studies.

To our knowledge, this is the first systematic review of patient education with a specific emphasis on abdominal surgery. We showed potential effects, current implementations, and frameworks of patient education in this systematic review. It is important to note that patient education is rarely an isolated intervention but rather part of a multimodal treatment with various concomitant interventions that may also affect the outcome. This leads to the fact that the educational interventions in the studies are hardly comparable. All but one study showed a reduced length of stay with integrated patient education, and some even showed a reduction in postoperative complications and anxiety. However, these results must be interpreted cautiously and are not directly transferable to clinical practice.

Nevertheless, patient education seems to have positive rather than negative effects on patients. None of the studies described adverse events due to patient education, except for higher pain in one study [23]. Thus, it can be assumed that patient education does not cause harm, provides the basis for communication at eye level, and encourages the patient to act in a participatory manner. Further studies in the field of patient education are necessary to be able to make concrete recommendations for clinical practice and, at the same time, to establish only meaningful measures in everyday clinical practice.
